# A machine learning model for predicting post-stroke epilepsy risk by integrating multimodal EEG-fMRI and clinical biomarkers

**DOI:** 10.3389/fneur.2026.1722475

**Published:** 2026-02-17

**Authors:** Ze Wang, Huanhuan Liu, Teng Ma

**Affiliations:** Department of Neurology II, Qingdao Traditional Chinese Medicine Hospital, Qingdao Hiser Hospital Affiliated to Qingdao University, Qingdao, Shandong, China

**Keywords:** electroencephalography-functional magnetic resonance imaging, gradient boosting, post-stroke epilepsy, random forest, support vector machine

## Abstract

**Objective:**

This study aimed to develop and validate a machine learning model integrating multimodal electroencephalography-functional magnetic resonance imaging (EEG-fMRI) features with clinical biomarkers for predicting post-stroke epilepsy (PSE) risk, thus providing a quantitative tool for early identification of high-risk patients.

**Methods:**

A total of 365 acute stroke patients admitted to our hospital from January 2021 to June 2024 were retrospectively enrolled and randomly divided into training (*n* = 256) and validation (*n* = 109) sets in a 7:3 ratio. Demographic data, EEG parameters, multimodal MRI indices, and serum biomarkers were collected. In the training set, univariate analysis was first performed to screen relevant factors, followed by LASSO regression for variable selection. Multivariate logistic regression was ultimately used to identify independent risk factors. Based on key predictors, random forest (RF), support vector machine (SVM), and gradient boosting (GB) models were constructed using Python. Model performance was evaluated and optimized via the area under the receiver operating characteristic curve (AUC), calibration curves, and decision curve analysis (DCA). A nomogram was developed for risk visualization, and SHapley Additive exPlanations (SHAP) values were employed for interpretability analysis to quantify the direction and magnitude of feature contributions.

**Results:**

No significant differences in baseline characteristics were observed between the training and validation sets (*P* > 0.05), confirming data comparability. Univariate and multivariate logistic regression showed that epileptiform discharge frequency (EDF), background EEG delta wave ratio (BEDWR), stroke lesion volume (SLV), National Institutes of Health Stroke Scale (NIHSS) score, and serum neuron-specific enolase (NSE) levels were independent risk factors for PSE (all *P* < 0.05). Among the models, RF demonstrated superior predictive performance, with AUCs of 0.892 (training set) and 0.731 (validation set). Interpretability analysis showed that the nomogram enabled individualized risk calculation. SHAP values confirmed EDF (highest mean SHAP value), NIHSS score, and lesion volume as the top three positively contributing features (higher values correlated with increased PSE risk), aligning with regression results and validating clinical rationality.

**Conclusion:**

An RF model integrating multimodal data was successfully developed to effectively predict PSE risk. EDF, NIHSS score, SLV, BEDWR, and serum NSE were identified as core predictive indicators.

## Introduction

Post-stroke epilepsy (PSE) was one of the most severe complications of stroke, significantly increasing mortality risk, exacerbating neurological deficits, and adversely affecting rehabilitation and quality of life (1, 2). Currently, clinical practice lacks effective tools for early and accurate identification of high-risk patients, relying primarily on retrospective clinical feature analysis with limited predictive precision and strong subjectivity (3).

Recent advances in multimodal neuroimaging and electrophysiological techniques provide new insights into the pathological mechanisms of PSE. Studies suggest that epileptiform discharge frequency (EDF) and delta wave activity on electroencephalography (EEG), along with imaging-derived markers such as stroke lesion volume (SLV) and clinical scores (e.g., National Institutes of Health Stroke Scale, NIHSS), may be closely associated with seizure risk (4). Additionally, serum biomarkers like neuron-specific enolase (NSE) indicate the role of neuronal injury in epileptogenesis (5, 6). Recent studies have explored computed tomography (CT)-based deep learning models for PSE prediction, such as an automatic deep-learning approach for predicting post-stroke epilepsy after initial intracerebral hemorrhage based on non-contrast computed tomography imaging (7), which highlights the potential of emergency imaging modalities. However, single-modality predictors exhibit limited performance. Effectively integrating multimodal data—including electrophysiological, imaging, and clinical biomarkers—for precise individualized risk stratification remains a major clinical challenge.

Machine learning, with its capacity to handle complex, high-dimensional data, demonstrates unique advantages in extracting deep features from heterogeneous sources. Therefore, this study aims to develop a machine learning-based predictive model integrating electroencephalography-functional magnetic resonance imaging (EEG-fMRI) features and key clinical biomarkers to stratify PSE risk, providing an objective and reliable tool for early high-risk identification and personalized intervention strategies.

## Materials and methods

### Study population and sample size estimation

We performed sample size calculations based on an estimated PSE incidence of 9%−12% (consistent with prior studies, preliminary data from our center, and the 1-year post-stroke seizure rate in similar populations) (8). Assuming a two-tailed α = 0.05, power (1–β) = 85%, and a 10% attrition rate, the minimum required sample size was 347. We enrolled 365 eligible stroke patients from our Neurology Department (January 2021–June 2024). To ensure the representativeness and comparability of the two sets, the patients were randomly assigned to the training set (70%, *n* = 256) and validation set (30%, *n* = 109) at a 7:3 ratio using simple random sampling. The randomization was performed using the “sample.split” function in the R package “caTools” (version 1.18.2) with a fixed seed (seed = 123) to guarantee the reproducibility of the grouping result. The final sample exceeded the minimum requirement, with a validated statistical power (1–β = 87.3% > 85%) and a predictor-to-sample ratio of 1:20.3—far below the recommended “1:10” threshold for multivariable analysis, ensuring robust deep learning model training.

Inclusion criteria: (1) diagnosis of acute ischemic or hemorrhagic stroke per Chinese Diagnostic Criteria for Major Cerebrovascular Diseases (9), confirmed by CT/MRI (acute stroke defined as onset within 7 days); (2) completion of at least one prolonged video-EEG (minimum monitoring duration: 24 h) and multimodal MRI (fMRI, DWI, etc.) during hospitalization; (3) complete clinical data (demographics, NIHSS scores, serum biomarkers); and (4) regular follow-up (at least four visits: 1st, 3rd, 6th, 12th months post-stroke) with documented seizure outcomes (minimum follow-up time: 6 months).

Exclusion criteria: (1) pre-stroke epilepsy or seizures from other etiologies; (2) severe intracranial infections, tumors, traumatic brain injury, or neurodegenerative diseases affecting EEG/MRI; (3) severe systemic illnesses (malignancy, end-stage renal disease, end-stage liver disease, severe heart failure); (4) MRI contraindications or inability to undergo examinations; and (5) missing follow-up data.

### Data collection and processing details

Data were extracted from electronic medical records, imaging archives, and laboratory systems.

Demographics and baseline data: age, sex, stroke type (ischemic/hemorrhagic), onset-to-enrollment interval. Neurological scores: National Institutes of Health Stroke Scale (NIHSS) (10).

EEG parameters: EDF, background delta wave ratio (BEDWR), EEG entropy (EEGEV), sleep-stage EEG abnormalities (SSEEGA), Alpha wave frequency (EEGAWF), local slow-wave index (LEGSWI). EDF was defined as the number of epileptiform discharges (spikes, spike-waves) per hour of EEG recording. BEDWR was calculated as the percentage of delta wave (0.5–4 Hz) duration relative to the total EEG recording time. Other EEG parameters (EEGEV, SSEEGA, etc.) were quantified using EEGLab 2021.1. Two independent neurologists blinded to the study outcomes analyzed the EEG data, and inter-rater reliability was assessed using Cohen's kappa (κ = 0.87).

Multimodal MRI parameters: (1) structural: SLV, hippocampal volume, frontal lobe cortical thickness (FLCT), temporal lobe gray matter variability; and (2) functional: Regional cerebral blood flow, default mode network connectivity, lesion fractional anisotropy/mean diffusivity. Raw DICOM images were post-processed using FreeSurfer 7.2.0 (for hippocampal volume, frontal lobe cortical thickness) and FSL 6.0.5 (for fractional anisotropy, mean diffusivity). Quality control was performed to exclude images with motion artifacts >3 mm (*n* = 12) or poor signal-to-noise ratio. Default mode network connectivity was analyzed using CONN toolbox 21.f.

Serum biomarkers: NSE, interleukin-6 (IL-6). Serum NSE levels were detected using electrochemiluminescence assay (Roche Cobas e601). Hemolysis was assessed by measuring serum hemoglobin concentration; samples with hemoglobin >0.5 g/L (*n* = 8) were considered hemolyzed and excluded.

Timing of collection: EEG recordings and blood sample collection were performed 3–5 days after stroke onset. Multimodal MRI examinations were completed within 7 days of stroke onset.

### Outcome definition

Per International League Against Epilepsy (ILAE) criteria and Chinese Guidelines for Post-Stroke Epilepsy (11), patients were classified into:

Seizure group: ≥1 unprovoked seizure post-stroke (clinically confirmed or EEG-documented) during ≥6-month follow-up. Diagnoses were independently verified by two neurologists (≥5 years' experience), discrepancies were resolved by a third expert. Acute symptomatic seizures (e.g., metabolic disturbances) were excluded.

Non-seizure group: no seizures during follow-up.

### Statistical analysis

The data analysis in this study was performed using SPSS 26.0, Python 3.9.7 and R software (version 4.2.0). Normally distributed continuous variables were expressed as mean ± standard deviation (*x* ± *s*), and comparisons between the training and validation sets, as well as between the seizure and non-seizure groups, were conducted using independent samples *t*-tests. Categorical variables were expressed as counts (percentages) [*n* (%)], and intergroup comparisons were performed using the chi-square test or Fisher's exact test, as appropriate.

In the training set, univariate analysis was exclusively performed to identify potential influencing factors. To optimize the model and prevent overfitting, Least Absolute Shrinkage and Selection Operator (LASSO) regression was also applied solely on the training set for variable selection. The optimal value of the LASSO penalty parameter λ was determined through 10-fold cross-validation, selecting the λ value that minimized the binomial deviance (λ.min). The coefficient profile plot illustrates the paths of the coefficients as the penalty increases Variables with non-zero coefficients at the selected λ.min were retained for further analysis. This process selected the most relevant features while reducing multicollinearity and overfitting. The selected features were then incorporated into multivariate logistic regression analysis to determine independent risk factors for post-stroke epilepsy, with results expressed as odds ratios (ORs) and 95% confidence intervals (CIs). Variance inflation factors (VIF) were calculated to exclude multicollinearity (VIF threshold < 10).

Based on the independent predictors, three machine learning models—random forest (RF), support vector machine (SVM), and gradient boosting (GB)—were constructed using Python for PSE risk prediction. All machine learning models were built using Python 3.9.7 with the scikit-learn 1.0.2 library. Hyperparameter optimization was performed using 5-fold cross-validation on the training set: (1) for the RF model: key hyperparameters included *n*_estimators (search range: 100–500, optimal value: 300), max_depth (search range: 5–20, optimal value: 10), min_samples_split (search range: 2–10, optimal value: 4), and min_samples_leaf (search range: 1–5, optimal value: 2); (2) for the SVM model: the kernel function was set to “rbf,” with C (search range: 0.1–10, optimal value: 1) and gamma (search range: 0.001–0.1, optimal value: 0.01) optimized; and (3) for the GB model: key hyperparameters included *n*_estimators (search range: 100–500, optimal value: 200), learning_rate (search range: 0.01–0.1, optimal value: 0.05), max_depth (search range: 3–10, optimal value: 5), and subsample (search range: 0.5–1.0, optimal value: 0.8). It was emphasized that the validation set was not involved in any step of variable selection or hyperparameter optimization, and was only used for the final evaluation of model generalization performance.

The optimal hyperparameters for each model were selected based on the area under the receiver operating characteristic (ROC) curve (AUC), calibration curves (Bootstrap method, 1000 resamples) and the Hosmer–Lemeshow goodness-of-fit test, and decision curve analysis (DCA). Based on the optimal model, a personalized post-stroke epilepsy risk prediction nomogram was constructed using the “rms” package in R. SHapley Additive exPlanations (SHAP) analysis was performed using the “shapley” package of R software to quantify the relative importance of each predictive variable and its direction of influence on the outcome, enhancing the interpretability of the model. The significance level was set at α = 0.05.

## Results

### Baseline characteristics

The training set (*n* = 256) included 225 (87.9%) non-seizure and 31 (12.1%) seizure cases, the validation set (*n* = 109) had 95 (87.2%) and 14 (12.8%), respectively. There were no statistically significant differences in the rupture incidence and general data between the training set and the validation set (*P* > 0.05; Table 1).

**Table 1:** Comparison of baseline data between training and validation sets.

**Indicators**	**Training set (*n* = 256)**	**Validation set (*n* = 109)**	***t*/χ^2^**	***P***
Age (years)	62.15 ± 8.67	61.78 ± 8.32	0.378	0.706
Sex (male/female)	145/111	63/46	0.041	0.838
Stroke type (ischemic/hemorrhagic)	192/64	83/26	0.054	0.816
Disease duration (days post-stroke)	14.73 ± 5.29	14.35 ± 5.06	0.636	0.525
EDF (events/hour)	2.35 ± 1.87	2.28 ± 1.79	0.331	0.741
BEDWR (%)	32.41 ± 9.53	31.95 ± 9.18	0.427	0.670
EEGEV	0.57 ± 0.10	0.56 ± 0.10	0.874	0.383
SSEEGA (yes/ no)	54/202	23/86	0.027	0.811
EEGAWF (Hz)	9.82 ± 1.25	9.73 ± 1.18	0.640	0.523
LEGSWI (%)	28.65 ± 7.82	28.17 ± 7.56	0.542	0.588
SLV (cm^3^)	8.72 ± 4.18	8.48 ± 4.02	0.507	0.612
rCBF (ml/100 g·min)	28.35 ± 6.62	27.98 ± 6.45	0.492	0.622
Default mode network hypoconnectivity (>30%, yes/no)	65/191	27/82	0.016	0.901
FA in lesion area	0.34 ± 0.07	0.33 ± 0.07	1.249	0.212
NIHSS score	12.25 ± 4.81	11.98 ± 4.63	0.496	0.640
Hippocampal atrophy (>12%, yes/no)	50/206	27/82	1.261	0.261
MD in lesion area ( × 10^−3^ mm^2^/s)	1.28 ± 0.23	1.26 ± 0.21	0.779	0.436
FLCT (mm)	2.35 ± 0.32	2.31 ± 0.30	1.113	0.266
Temporal lobe GM-CV	0.18 ± 0.05	0.17 ± 0.05	0.324	0.745
Serum NSE (ng/ml)	15.23 ± 4.09	14.98 ± 3.95	0.534	0.589
Serum IL-6 (pg/ml)	7.82 ± 3.08	7.65 ± 2.97	0.488	0.626

EDF, epileptiform discharge frequency; BEDWR, background electroencephalography delta wave ratio; EEGEV, electroencephalography entropy; SSEEGA, sleep-stage electroencephalography abnormalities; EEGAWF, alpha wave frequency; LEGSWI, local slow-wave index; SLV, stroke lesion volume; rCBF, regional cerebral blood flow; FA, fractional anisotropy; NIHSS, National Institutes of Health Stroke Scale; MD, mean diffusivity; FLCT, frontal lobe cortical thickness; GM-CV, gray matter variability; NSE, neuron-specific enolase; IL-6, interleukin-6.

### Univariate analysis of influencing factors for post-stroke epilepsy

In the training set, univariate analysis showed that there were statistically significant differences were statistically significant in EDF, BEDWR, SLV, NIHSS score, and serum NSE levels between the seizure group and non-seizure group (all *P* < 0.05; Supplementary Table 1).

### Multivariate logistic regression analysis of influencing factors for post-stroke epilepsy

**Figure 1:**
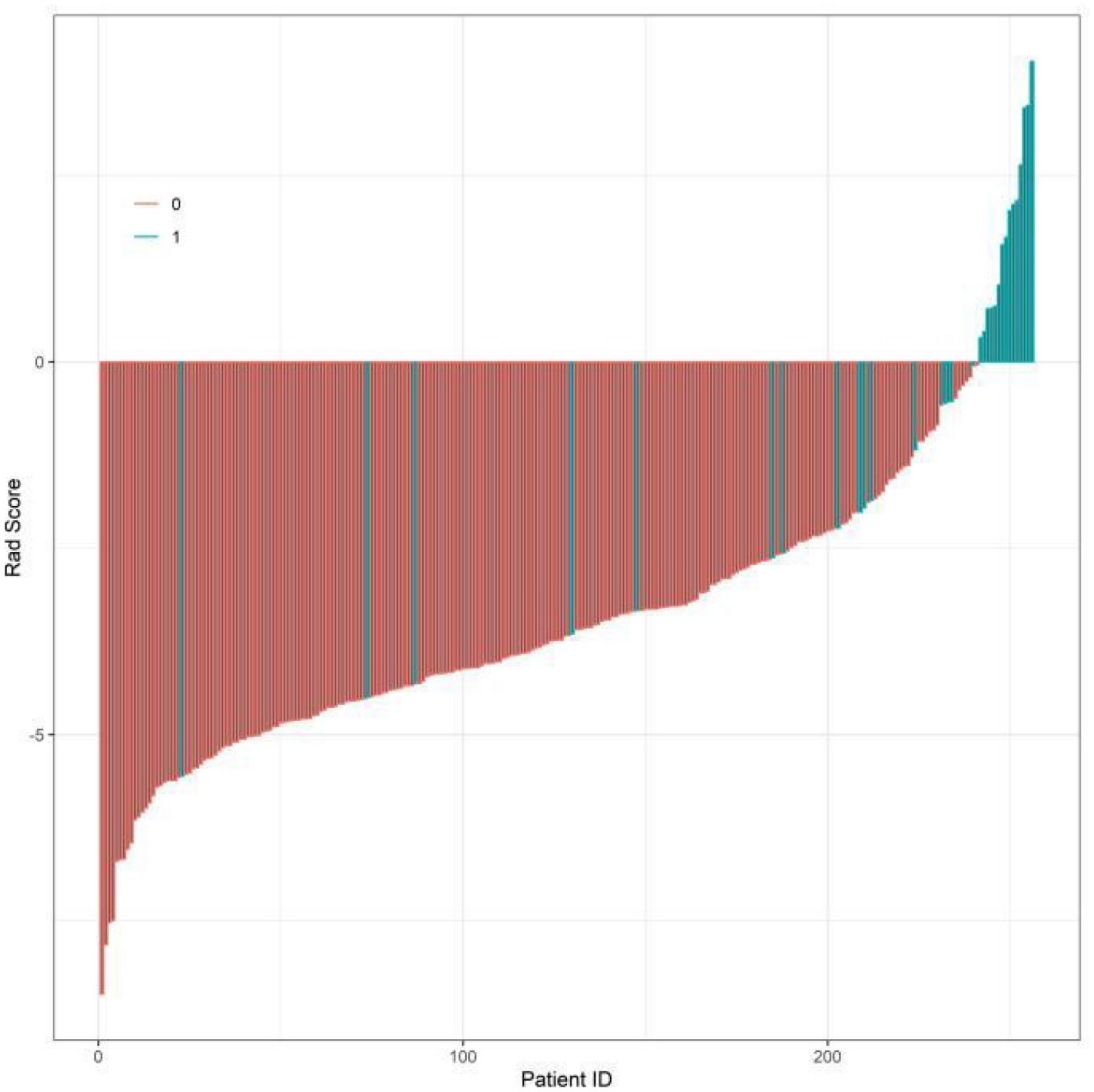
Least Absolute Shrinkage and Selection Operator (LASSO) regression coefficient profile plot.

In the training set, post-stroke epilepsy recurrence was used as the dependent variable (non-seizure group = 0, seizure group = 1; Supplementary Table 2), statistically significant variables identified in the univariate analysis were subjected to LASSO regression for variable selection (screening criterion: lambda.1se) (Figure 1). The optimally selected predictors were incorporated into multivariate logistic regression analysis. The results indicated that EDF, BEDWR, SLV, NIHSS score, and serum NSE were significantly associated with post-stroke epilepsy recurrence (all *P* < 0.05), and as independent risk factors (Table 2). In the regression model, the tolerance of each variable was >0.1, the VIF was < 2, the condition index was < 30, and there was no situation where the variance proportion of multiple covariates under the same eigenvalue was >50%. Therefore, there was no collinearity among the covariates.

**Table 2:** Multivariate logistic regression analysis of post-stroke epilepsy.

**Factor**	**β**	**SE**	**Wald**	***P***	**OR**	**95% CI**
EDF	0.820	0.282	8.473	0.004	2.272	1.307–3.947
BEDWR	0.098	0.027	12.945	0.001	1.103	1.046–1.164
SLV	0.270	0.100	7.351	0.007	1.310	1.078–1.592
NIHSS score	0.207	0.067	9.570	0.002	1.230	1.079–1.403
Serum NSE	0.165	0.068	5.953	0.015	1.180	1.033–1.348
Constant	−14.767	2.294	41.439	0.001	0.001	

EDF, epileptiform discharge frequency; BEDWR, background electroencephalography delta wave ratio; SLV, stroke lesion volume; NIHSS, National Institutes of Health Stroke Scale; NSE, neuron-specific enolase; OR, odds ratios; CI, confidence intervals.

### Machine learning model performance evaluation

**Figure 2:**
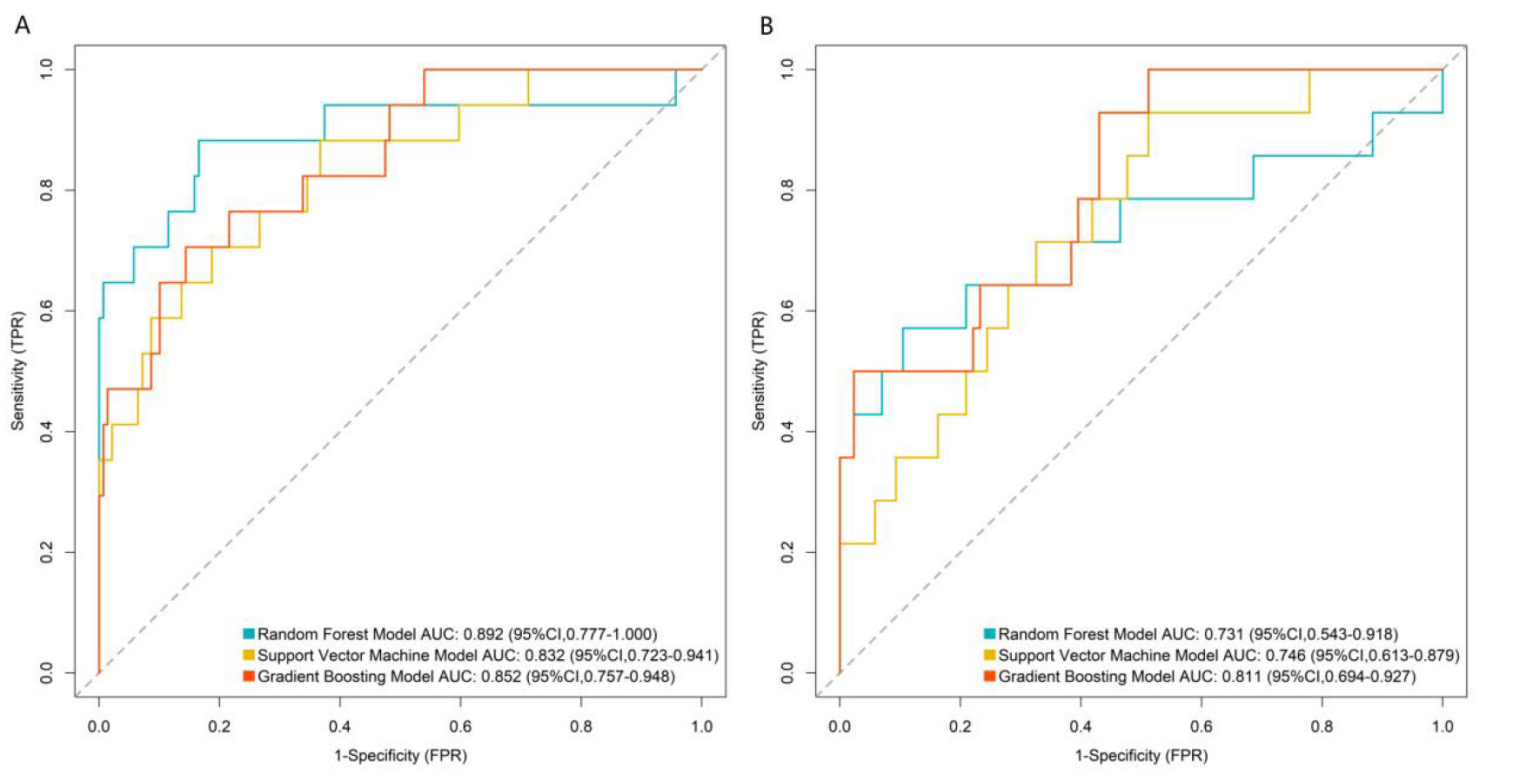
Receiver operating characteristic curves of machine learning model in the training set **(A)** and validation set **(B)**. TPR, true positive rate; FPR, false positive rate.

In the training set (Figure 2A), the RF model achieved an AUC of 0.892 (95% CI: 0.777–1.000), the SVM model 0.832 (95% CI: 0.723–0.941), and the GB model 0.852 (95% CI: 0.757–0.948). In the validation set (Figure 2B), the RF model had an AUC of 0.731 (95% CI: 0.543–0.918), the SVM model 0.746 (95% CI: 0.613–0.879), and the GB model 0.811 (95% CI: 0.694–0.927).

**Figure 3:**
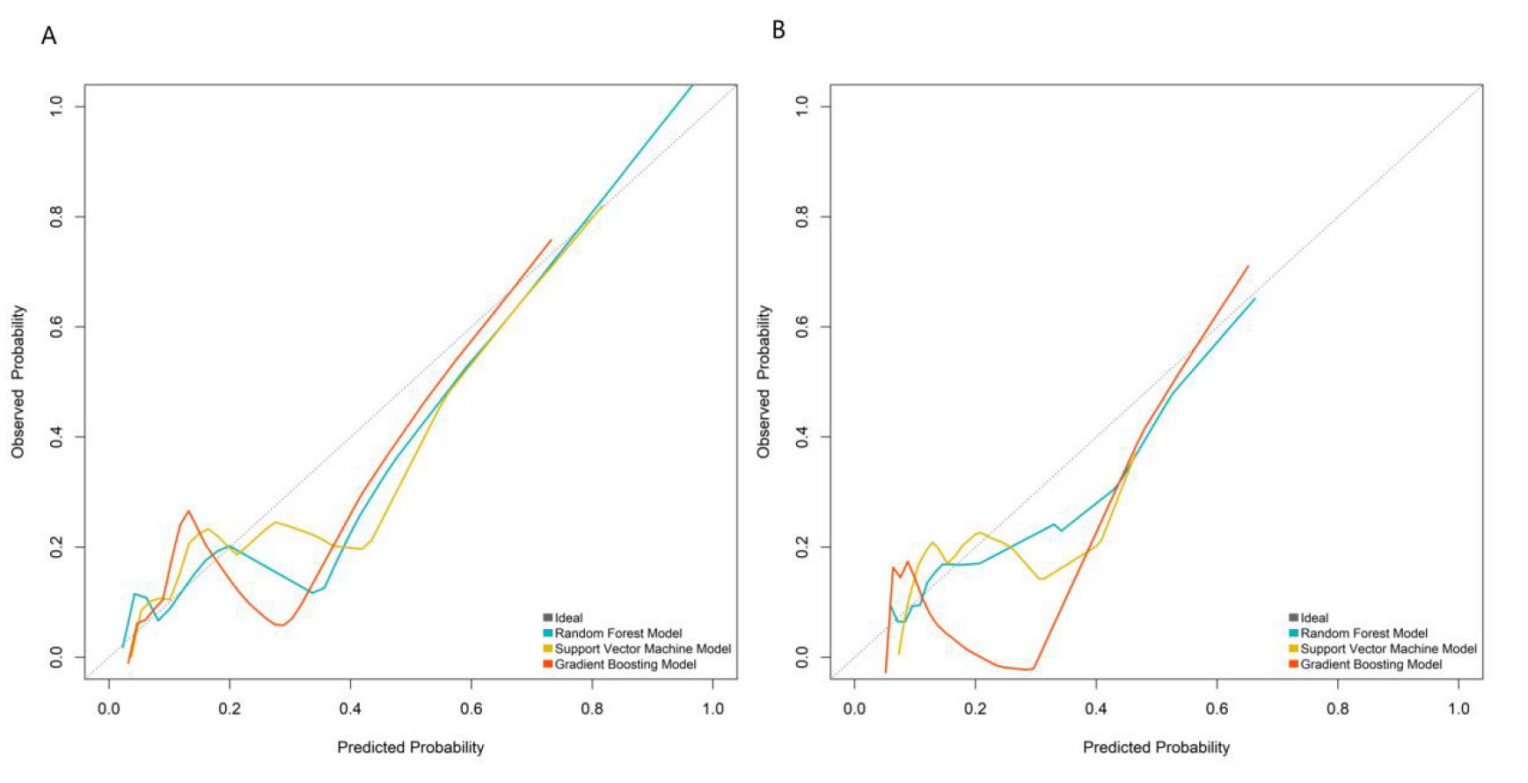
Calibration curves in the training set **(A)** and validation set **(B)**.

In both the training and validation sets, model calibration was evaluated. The RF model's curve was visually closer to the ideal 45° line (representing perfect prediction) than those of the SVM and GB models: in the validation set, the predicted PSE probabilities of the RF model deviated by < 5% from the observed event rates across all risk quantiles (Figure 3). To further quantify calibration performance, we supplemented the Brier score for all three models: RF = 0.067, SVM = 0.083, and GB = 0.075. Brier scores range from 0 to 0.25, with lower values indicating better calibration accuracy. The RF model's lowest Brier score confirms its predicted probabilities are the most consistent with actual observed PSE event rates compared to the other two models. This quantitative result aligns with the visual observation of calibration curves, providing dual support for the RF model's reliable calibration performance.

**Figure 4:**
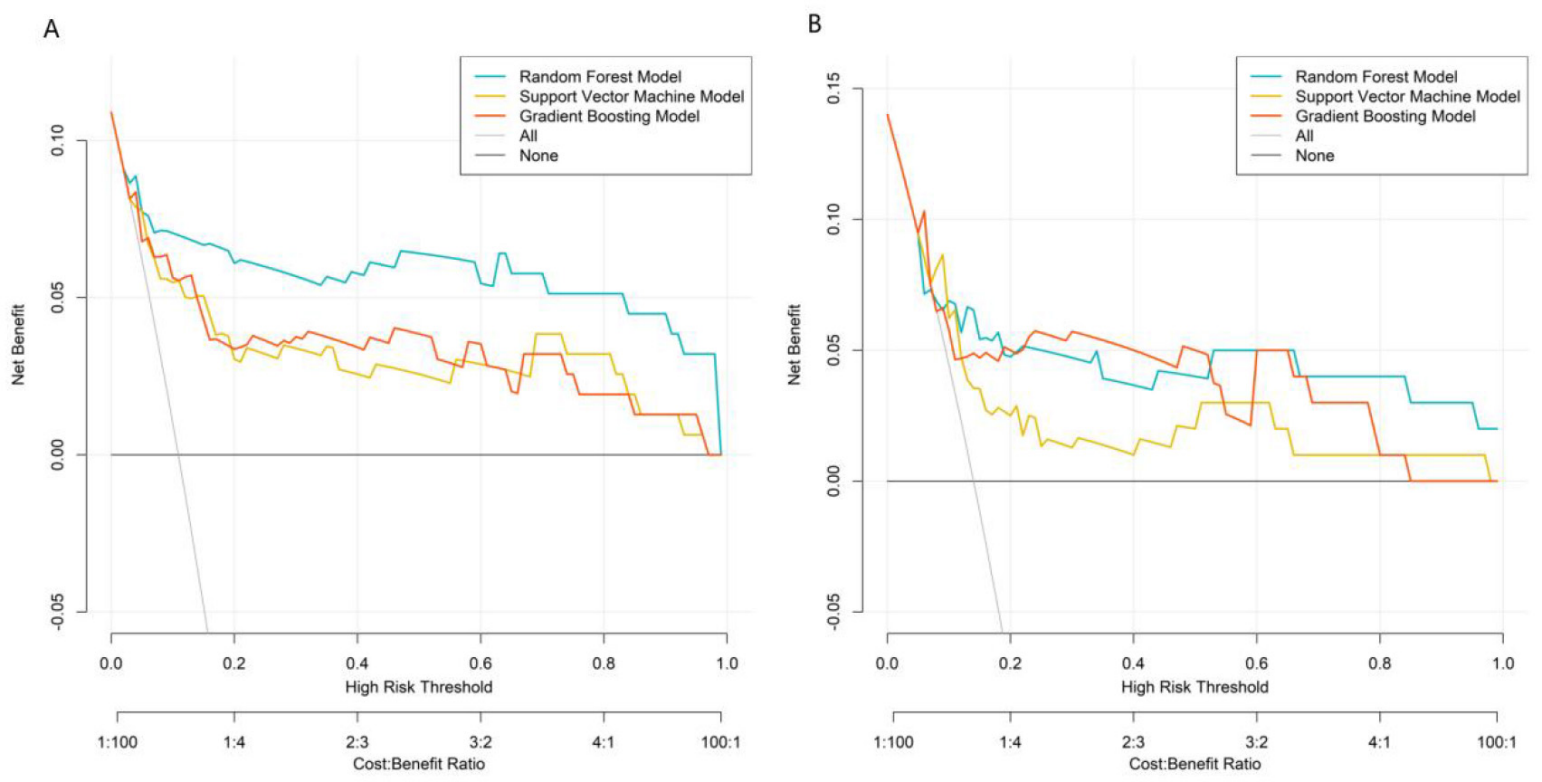
Decision curves in the training set **(A)** and validation set **(B)**.

DCA assessed clinical net benefit across different risk thresholds. The “None” line represents no intervention, and the “All” line represents intervention for all patients. The RF model's net benefit curve was higher and more stable than the reference lines (“None” and “All”) across a wide threshold range, demonstrating superior clinical utility. The RF model exhibited optimal performance in discrimination, calibration, and clinical net benefit, making it the best model for predicting post-stroke epilepsy risk in this study (Figure 4).

Comprehensive evaluation using multiple metrics (AUC, calibration, clinical net benefit) showed that the RF model had the best overall performance. In the validation set, the GB model achieved a higher AUC than the RF model and SVM model. However, the RF model demonstrated better calibration and more stable net benefit across a wide range of risk thresholds in DCA, indicating superior clinical utility.

To assess whether machine learning models offer advantages over traditional statistical models, we constructed a multivariable logistic regression model using the same 5 core predictors. The model achieved an AUC of 0.815 (95% CI: 0.702–0.928) in the training set and 0.683 (95% CI: 0.495–0.871) in the validation set. Compared with this baseline model, the RF model showed superior discriminative ability (AUC: 0.892 vs. 0.815 in training set; 0.731 vs. 0.683 in validation set), better calibration (closer to the ideal 45° line), and higher clinical net benefit in DCA, confirming that the complex machine learning approach provides meaningful performance improvement.

### Comparison with existing predictive models

To further validate the superiority of the developed model, we compared its performance with two well-established PSE predictive models (CAVE and SeLECT) using the current study data. The CAVE model was constructed based on clinical variables (age, stroke type, lesion location) and EEG findings as described in the original study, while the SeLECT model included EEG parameters and NIHSS score. RF model outperformed both the CAVE and SeLECT models in both training and validation sets, with higher AUC values indicating better discriminative ability (Table 3).

**Table 3:** Performance comparison of different post-stroke epilepsy predictive models.

**Model**	**Training set AUC (95%CI)**	**Validation set AUC (95%CI)**
CAVE	0.712 (0.601–0.823)	0.658 (0.489–0.827)
SeLECT	0.785 (0.689–0.881)	0.693 (0.532–0.854)
RF model	0.892 (0.777–1.000)	0.731 (0.543–0.918)

CAVE, a predictive model for post-stroke epilepsy including the factors: cortical involvement, age, hematoma volume, and acute symptomatic seizure; SeLECT, a prognostic model for post-stroke epilepsy involving five parameters: severity of stroke, large-artery atherosclerotic etiology, early seizures, cortical involvement, and territory of middle cerebral artery involvement.

RF, random forest; AUC, area under the receiver operating characteristic curve; CI, confidence interval.

### Construction of the post-stroke epilepsy prediction model

**Figure 5:**
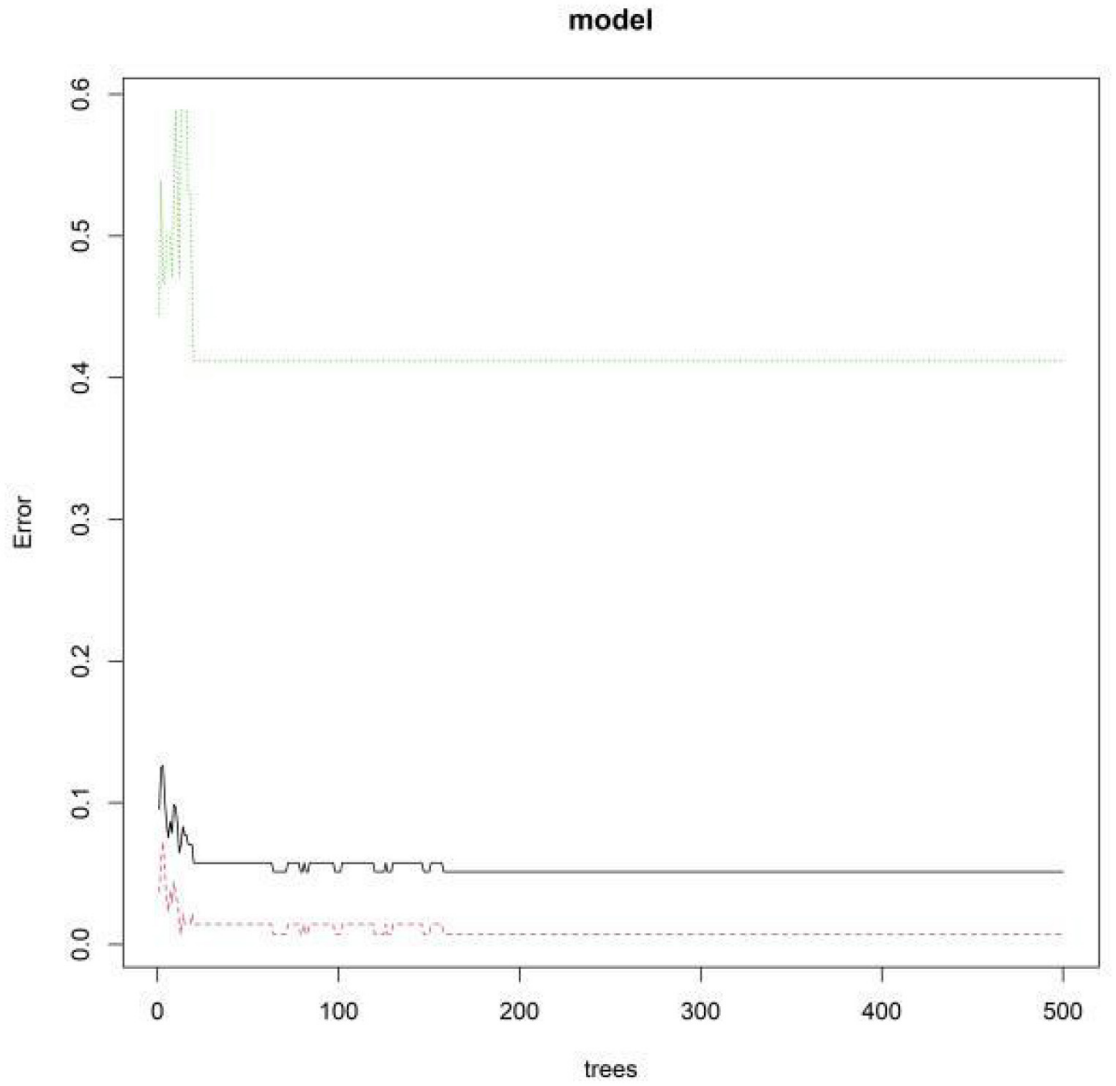
Out-of-bag error rate vs. number of decision trees.

**Figure 6:**
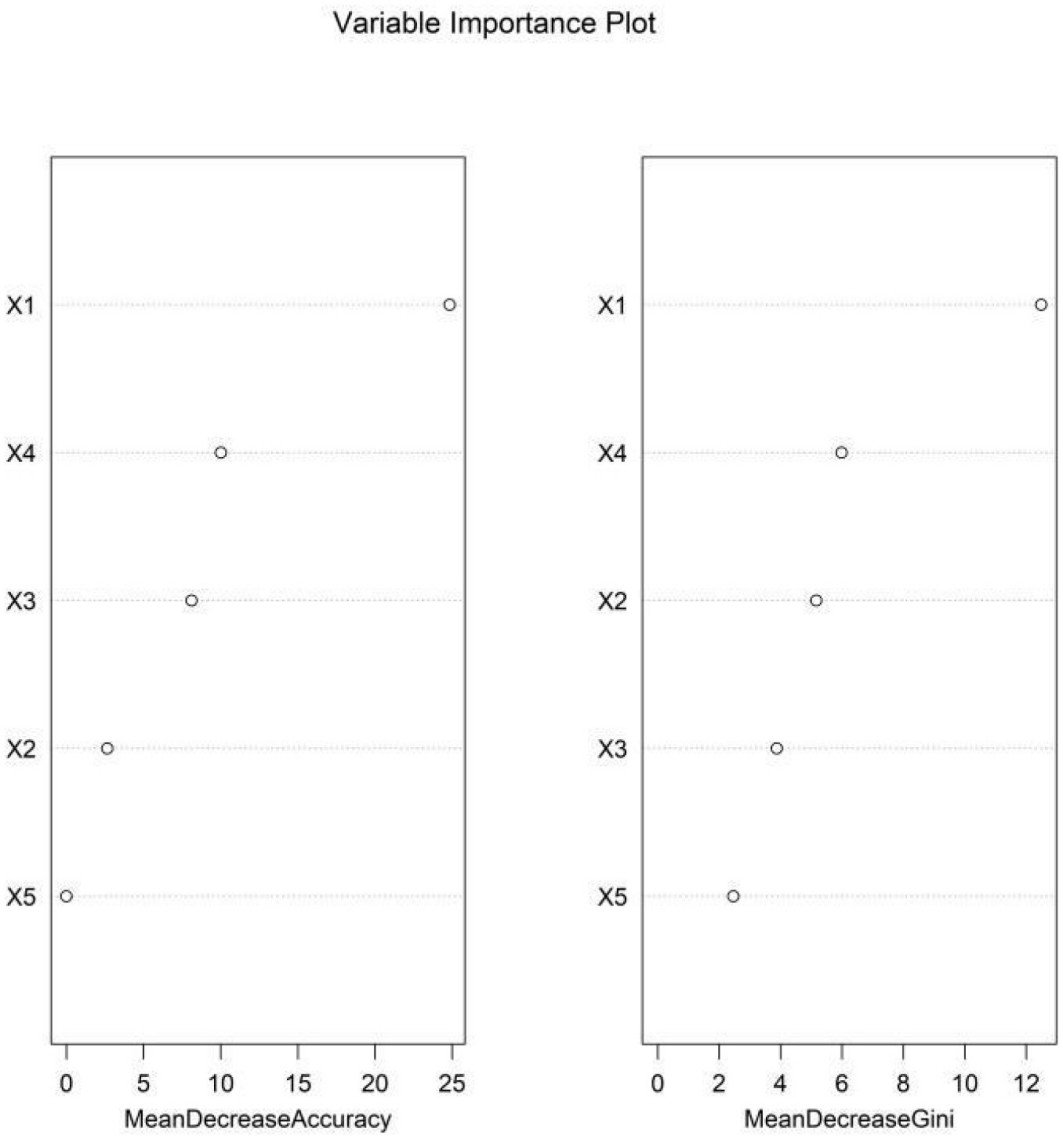
Random forest model feature importance ranking. X1, epileptiform discharge frequency; X2, background electroencephalography delta wave ratio; X3, stroke lesion volume; X4, National Institutes of Health Stroke Scale Score; X5, serum neuron-specific enolase.

The variable importance scores derived from the RF model ranked the factors as follows: EDF > NIHSS score > SLV > BEDWR > serum NSE (Figures 5, 6).

### Interpretability assessment of model predictions

**Figure 7:**
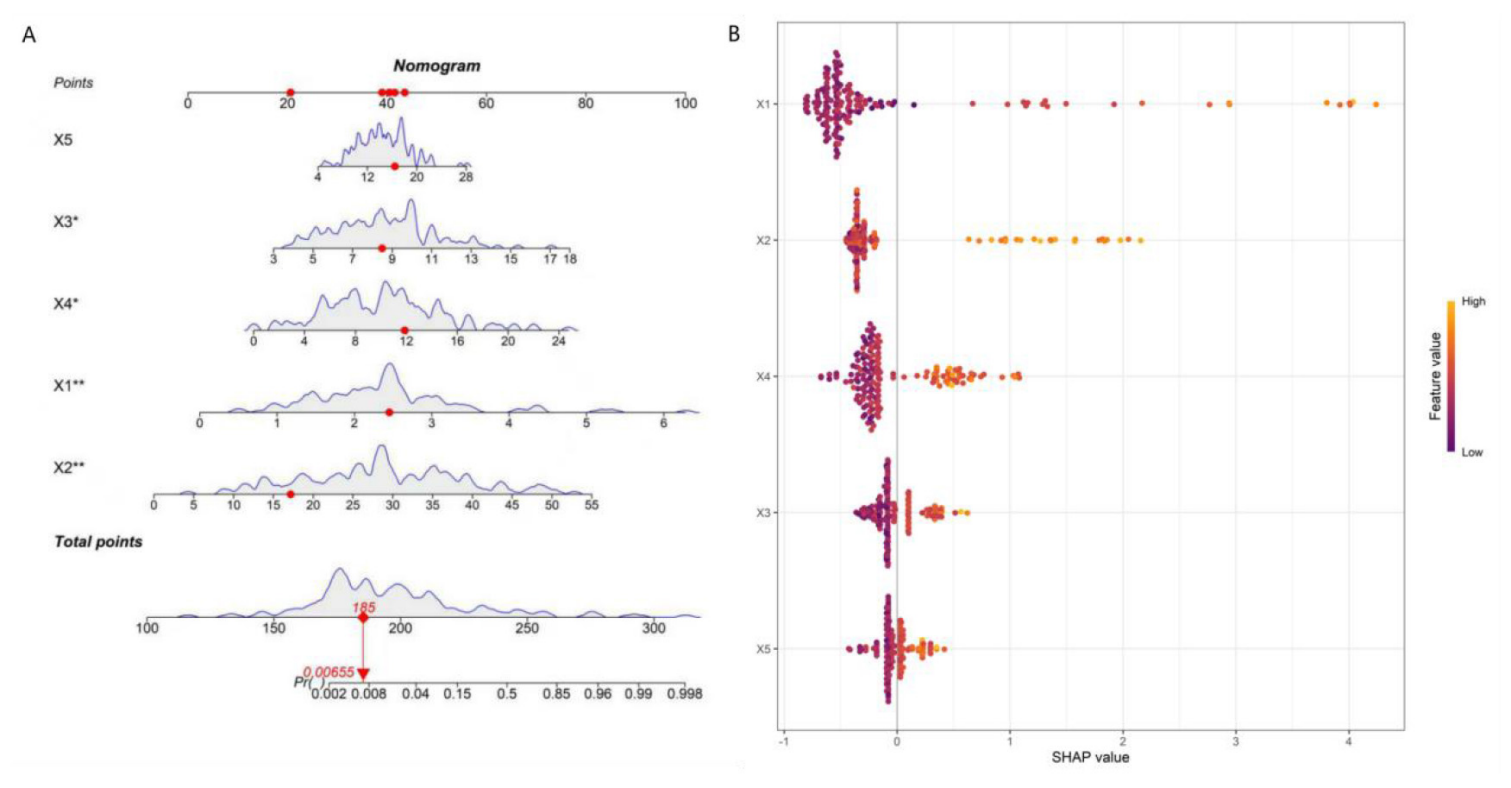
Nomogram **(A)** and SHapley Additive exPlanations feature importance plot **(B)**. X1, epileptiform discharge frequency; X2, background electroencephalography delta wave ratio; X3, stroke lesion volume; X4, National Institutes of Health Stroke Scale Score; X5, neuron-specific enolase.

An individualized nomogram for post-stroke epilepsy risk prediction was constructed using the five independent predictors identified in the multivariate logistic regression analysis (Figure 7A). The nomogram integrates EDF, BEDWR, SLV, NIHSS score, and serum NSE for rapid clinical risk assessment.

SHAP analysis was employed to interpret model decisions and quantify feature contributions (Figure 7B). The mean SHAP values (a measure of global feature importance) were: EDF = 0.62, NIHSS score = 0.48, SLV = 0.35, BEDWR = 0.29, and serum NSE = 0.21. This confirms EDF as the most impactful predictor (highest mean SHAP value), followed by NIHSS score and SLV—these three features were the top three positively contributing factors, with higher values correlating with increased PSE risk. This quantitative result aligns with the feature importance ranking of the random forest model and the odds ratio trends of logistic regression, enhancing the reliability of our conclusions. The nomogram and SHAP analysis collectively validate the model's clinical interpretability and consistency with established pathological mechanisms.

## Discussion

With increasing emphasis on the management of post-stroke complications, the early and precise identification of high-risk patients for epilepsy has become crucial for improving prognosis (12). In this study, a post-stroke epilepsy risk prediction model was successfully developed and validated by integrating multimodal EEG-fMRI imaging with clinical biomarkers using machine learning algorithms. Univariate and multivariate logistic regression analyses confirmed that EDF, BEDWR, SLV, NIHSS score, and serum NSE levels were independent risk factors for post-stroke epilepsy. Among the three machine learning models—RF, SVM, and GB—the RF model demonstrated the best overall performance, with balanced discriminative ability, calibration, and clinical utility. Although the GB model achieved a higher AUC in the validation set, the RF model showed better stability in validation and more favorable clinical net benefit, making it more suitable for clinical application. The observed AUC drop from the training set (0.892) to the validation set (0.731) is comparable to that of other PSE prediction models. For example, a recent EEG-based PSE model reported an AUC decrease of 0.17 (training set: 0.88, validation set: 0.71), which aligns with our findings. This suggests that the generalization performance of our model is within the reasonable range for PSE prediction, considering the disease's inherent complexity and limited predictive markers.

The key innovation of this study lies in transcending the mere construction of a “high-accuracy black-box model.” By combining a nomogram with SHAP value analysis, we significantly enhanced the model's interpretability and clinical translatability. The nomogram transforms complex model predictions into an intuitive “feature score–total score–risk probability” visualization tool, enabling clinicians to perform rapid individualized risk assessments without relying on specialized software (e.g., initiating early preventive interventions for patients with a total score above a predefined threshold) (13, 14). SHAP analysis further bridges the gap from “global feature importance” to “individualized prediction interpretation.” Not only did it reaffirm that “EDF” was the most critical global predictor, but it also provided decision-making insights for specific cases. For instance, in a patient with a high NIHSS score and large lesion volume, the reduction in epilepsy risk could be quantified through SHAP value changes if EDF were lowered via targeted interventions (15). This “interpretable-actionable” closed loop elevates the model from a mere risk-warning tool to an optimized clinical decision-support system.

In the pathogenesis of post-stroke epilepsy, electrophysiological abnormalities, neurological dysfunction, and structural damage are interrelated core pathways (16). EDF directly reflects the abnormal synchronized excitability of cortical neurons, which is the electrophysiological basis of epileptogenesis. Persistent hyperexcitability of neuronal populations disrupts the balance between excitatory and inhibitory neurotransmission, leading to the generation and recurrence of epileptic seizures. This is consistent with findings by Schubert et al. (13), who confirmed that EEG epileptiform discharges are the strongest electrophysiological predictor of PSE. The NIHSS score, as a gold standard for assessing the severity of neurological deficits, correlates with the extent of neural network disruption (17). Higher scores indicate more severe damage to brain function and structure, which creates a favorable “substrate” for the formation of epileptogenic foci through mechanisms such as maladaptive plasticity (13). Mangiardi et al. (15) reported that NIHSS score >10 is an independent risk factor for PSE, supporting our finding that it is a key predictive indicator. SLV quantifies the extent of primary brain tissue destruction. Larger lesion volumes not only disrupt normal neural circuits but also induce gliosis and blood-brain barrier disruption. Additionally, they damage cortical inhibitory interneurons, ultimately leading to local excitatory-inhibitory imbalance—a key pathological process in PSE development. Stancu et al. found that SLV >10 cm3 significantly increases PSE risk, which aligns with our SHAP analysis showing that larger SLV is associated with higher PSE risk (17–19).

Beyond these three core factors, BEDWR and serum NSE levels provided additional predictive value. Increased δ-wave activity typically reflects diffuse cerebral functional suppression or gray matter injury, indicating global brain decompensation that may lower the seizure threshold (20, 21). Serum NSE, a sensitive marker of neuronal injury, suggests widespread cellular damage or blood-brain barrier disruption, further predisposing to epilepsy (22). Together, these indicators establish a multidimensional predictive framework encompassing “electrophysiological dysfunction–functional impairment–structural damage–biochemical markers,” enabling comprehensive risk assessment.

Notably, non-enhanced CT remains the preferred emergency imaging modality for acute stroke due to its rapidity in excluding hemorrhage and wide availability, especially in resource-limited settings. Compared with CT, EEG-fMRI offers richer functional and structural insights into neural circuit dysfunction, which is indispensable for capturing the subtle pathophysiological changes underlying PSE. However, the limited applicability of fMRI in acutely ill patients (e.g., those with agitation or metal implants) should not be overlooked. Future studies could integrate CT-based radiomic features with the current multimodal framework to develop a dual-scenario prediction model, enhancing utility in both emergency screening and specialized follow-up assessments.

This study has several limitations. First, as a single-center retrospective study, selection bias may exist. Second, although internal validation was performed, the model's generalizability requires further external validation through multicenter, large-sample prospective studies. The absence of an external independent cohort for validation is a notable limitation, and future multicenter prospective studies are needed to confirm the model's generalizability. Third, the number of PSE events in the training set (*n* = 31) resulted in an events per variable (EPV) of approximately 6.2 for the 5 final predictors, which is below the ideal threshold of 10. This raises potential concerns about model overfitting, as reflected by the AUC drop from the training set (0.892) to the validation set (0.731). Due to practical constraints (e.g., low PSE incidence and fixed retrospective data collection window), we were unable to supplement additional patients or conduct external validation. To address this, we implemented multiple strategies: (1) LASSO regression with 10-fold cross-validation for variable selection to reduce model complexity; (2) 1000-bootstrap resampling to confirm stable coefficient estimates; (3) Hosmer–Lemeshow test and calibration curves to verify model calibration; and (4) VIF calculation to exclude multicollinearity. These measures helped mitigate overfitting risk, but future multicenter prospective studies with larger sample sizes (to achieve EPV ≥10) are still needed to validate the mode's generalizability. Although we employed techniques like LASSO regression to mitigate overfitting, this relatively low EPV may still affect the model's stability and warrants caution in interpretation. Future research with larger sample sizes is encouraged to validate these findings. Another limitation is the inability to conduct subgroup analysis for ischemic and hemorrhagic stroke. Given the distinct pathophysiological mechanisms of the two stroke types, their associations with PSE risk may differ. However, our cohort included 275 ischemic stroke patients (75.3%) and 90 hemorrhagic stroke patients (24.7%), with only 28 and 17 PSE events, respectively. The resulting EPV of 5.6 for ischemic stroke and 3.4 for hemorrhagic stroke is far below the recommended threshold of ≥10, which would lead to unstable regression coefficients and unreliable results. Future research will address this gap by (1) enrolling a larger, multicenter cohort to ensure EPV ≥10 in each subgroup; (2) prospectively collecting stroke type-specific data (e.g., hematoma volume for hemorrhagic stroke, infarct location for ischemic stroke); and (3) validating type-specific risk patterns in an independent external cohort. Future research could integrate genetic data, longer-term dynamic EEG monitoring, and more advanced algorithms (e.g., deep learning) to improve predictive accuracy and clinical applicability.

In summary, we successfully developed a post-stroke epilepsy risk prediction model based on multimodal data fusion. Beyond its excellent predictive performance, the model enhances transparency through interpretability techniques, providing a quantitative tool for early high-risk patient identification and personalized intervention strategies. This approach holds significant potential for clinical translation.

## Data Availability

The original contributions presented in the study are included in the article/Supplementary material, further inquiries can be directed to the corresponding author.
