# Supplementary material for: A machine learning model for predicting post-stroke epilepsy risk by integrating multimodal EEG-fMRI and clinical biomarkers
Source: Front Neurol. 2026 Feb 17;17:1722475. doi: 10.3389/fneur.2026.1722475 (PMC12953071; doi:10.3389/fneur.2026.1722475)
Supplement: Supplementary file 1 [file Data_Sheet_1.docx]

**Supplemental Table 1.** Univariate analysis of influencing factors for post-stroke epilepsy.

| Indicators | Seizure Group  (n=31) | Non-Seizure Group  (n=225) | *t*/*χ²* | P |
| --- | --- | --- | --- | --- |
| Age (years) | 60.15±8.67 | 62.65±8.87 | 1.475 | 0.141 |
| Sex (Male/Female) | 19/12 | 126/99 | 0.315 | 0.577 |
| Stroke Type (Ischemic/Hemorrhagic) | 24/7 | 168/57 | 0.110 | 0.740 |
| Disease Duration (days post-stroke) | 15.73±5.29 | 14.12±5.02 | 1.663 | 0.097 |
| EDF (events/hour) | 3.1±1.50 | 2.2±0.6 | 6.151 | 0.001 |
| BEDWR (%) | 38.41±9.53 | 28.41±9.53 | 5.477 | 0.001 |
| EEGEV | 0.58±0.10 | 0.57±0.01 | 1.465 | 0.144 |
| SSEEGA (Yes/ No) | 7/26 | 47/176 | 0.003 | 0.986 |
| EEGAWF (Hz) | 9.92±1.35 | 9.80±1.01 | 0.593 | 0.554 |
| LEGSWI (%) | 29.65±7.82 | 28.05±7.72 | 1.080 | 0.281 |
| SLV (cm³) | 9.97±3.26 | 8.32±2.14 | 3.743 | 0.001 |
| rCBF (mL/100g·min) | 28.38±6.62 | 28.31±6.00 | 0.060 | 0.952 |
| Default Mode Network Hypoconnectivity (>30%, Yes/ No) | 8/23 | 57/168 | 0.443 | 0.505 |
| FA in Lesion Area | 0.34±0.08 | 0.35±0.01 | 1.797 | 0.073 |
| NIHSS Score | 14.25±4.81 | 9.55±4.01 | 5.965 | 0.001 |
| Hippocampal Atrophy (>12%, Yes/ No) | 6/26 | 44/180 | 0.014 | 0.905 |
| MD in Lesion Area (×10⁻³ mm²/s) | 1.31±0.20 | 1.27±0.21 | 0.995 | 0.318 |
| FLCT (mm) | 2.36±0.33 | 2.34±0.31 | 0.334 | 0.739 |
| Temporal Lobe GM-CV | 0.19±0.04 | 0.17±0.06 | 1.800 | 0.073 |
| Serum NSE (ng/mL) | 17.23±4.55 | 14.23±4.01 | 6.400 | 0.001 |
| Serum IL-6 (pg/mL) | 7.85±3.11 | 7.80±3.01 | 0.086 | 0.931 |

**Note:** EDF, Epileptiform discharge frequency; BEDWR, Background Electroencephalography delta wave ratio; EEGEV, Electroencephalography entropy; SSEEGA, Sleep-stage Electroencephalography abnormalities; EEGAWF, Alpha wave frequency; LEGSWI, Local slow-wave index; SLV, Stroke lesion volume; rCBF, Regional cerebral blood flow; FA, Fractional anisotropy; NIHSS, National Institutes of Health Stroke Scale; MD, Mean diffusivity; FLCT, Frontal lobe cortical thickness; GM-CV, Gray matter variability; NSE, Neuron-specific enolase; IL-6, Interleukin-6.

**Supplemental Table 2.** Variable Assignments.

| Variable | Meaning | Assignment |
| --- | --- | --- |
| X1 | EDF | Continuous |
| X2 | BEDWR | Continuous |
| X3 | SLV | Continuous |
| X4 | NIHSS score | Continuous |
| X5 | Serum NSE | Continuous |
| Y | Seizure occurrence | Non-seizure group=0, seizure group=1 |

**Note**: EDF, Epileptiform discharge frequency; BEDWR, Background Electroencephalography delta wave ratio; SLV, Stroke lesion volume; NIHSS, National Institutes of Health Stroke Scale; NSE, Neuron-specific enolase.
